# A rare case of intraventricular gangliocytoma

**DOI:** 10.1590/0004-282X-ANP-2021-0261

**Published:** 2022-01-31

**Authors:** João Antonio Pessôa Corrêa, Lucas Teixeira Diniz, João Norberto Stavale, Felipe Campos Kitamura, Luis Antonio Tobaru Tibana, Márcio Luís Duarte, Leonardo Furtado Freitas

**Affiliations:** 1 Universidade Federal de São Paulo, Departamento de Radiologia, São Paulo SP, Brazil. Universidade Federal de São Paulo Departamento de Radiologia São Paulo SP Brazil; 2 Universidade Federal de São Paulo, Departamento de Patologia, São Paulo SP, Brazil. Universidade Federal de São Paulo Departamento de Patologia São Paulo SP Brazil; 3 Universidade Federal de São Paulo, Departamento de Saúde Baseada em Evidências, São Paulo SP, Brazil. Universidade Federal de São Paulo Departamento de Saúde Baseada em Evidências São Paulo SP Brazil

**Figure 1.**
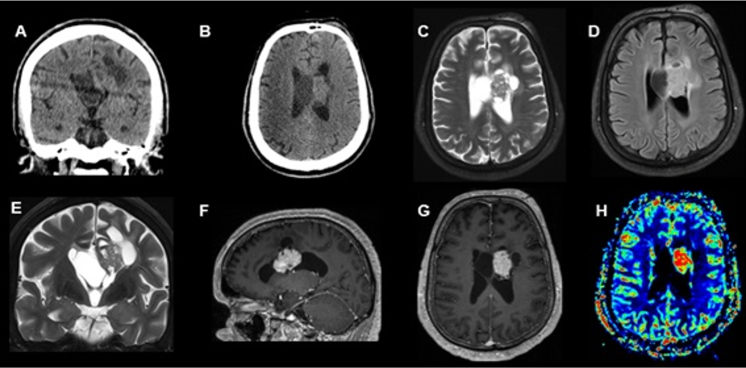
Coronal (A) and axial (B) nonenhanced brain CT shows a solid and cystic lesion within the lateral left ventricle and infiltrating adjacent white matter. Coronal and axial T2 (C, D), axial FLAIR (E) shows a well-demarcated, isointense and heterogeneous lesion with predominantly peripheral cysts located within the left lateral ventricle with infiltration of its lateral wall and the septum pellucidum. Sagittal and axial post-contrast T1 (F, G) sequence shows intense enhancement of the solid portion and increased rCBV on DSC perfusion (H).

FLAIR: fluid attenuated inversion recovery; rCBV: relative cerebral blood volume; DSC: differential scanning calorimetry.

**Figure 2.**
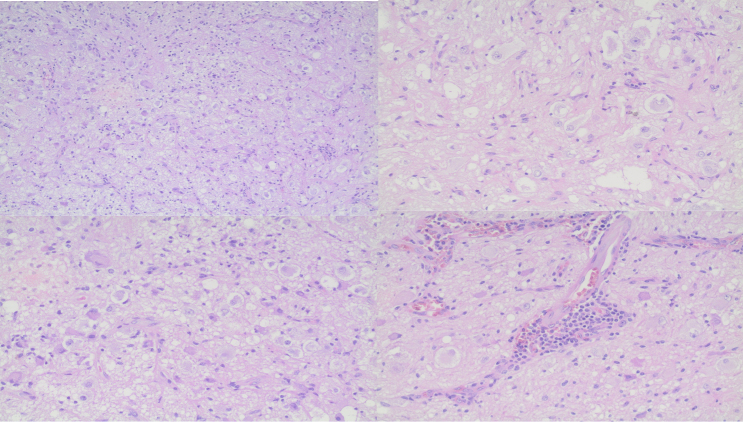
Clusters of atypical pleomorphic ganglion cells embedded in a haphazard manner within a delicate neuropil matrix. No neoplastic glial cells are present.

**Figure 3.**
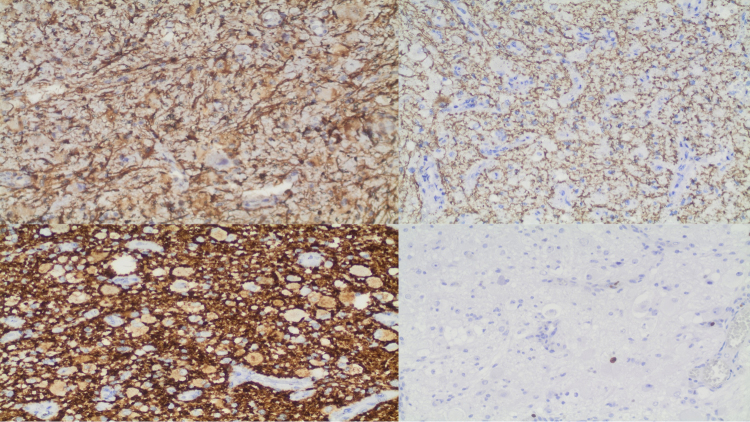
Immunohistochemical (IHC) studies. Glial fibrillary acidic protein (GFAP) positive in normal astrocytes with positive ATRX and negative isocitrate dehydrogenase. Neurofilament (NF) and synaptophysin (SYN) were positive in the neuropil. Ki-67 (Ki67), which determines the proliferative index, was low (< 2%).

A 49-year-old man presented to the emergency department after cranioencephalic trauma with an intraventricular tumor detected in the computed tomography (CT) scan (Figure 1). An magnetic resonance image (MRI) showed a heterogeneous expansive lesion with enhancing solid components and peripheral cysts located in the left lateral ventricle (Figure 1). The patient underwent excision of the lesion. Histopathologic (Figure 2) and immunohistochemical (Figure 3) analysis revealed the diagnosis of gangliocytoma. Gangliocytomas are rare low-grade central nervous system tumors composed of dysplastic ganglion cells, usually presenting in children or young adults and located in the cerebral hemispheres^1^^,^^2^. Until now, there are no case reports of intraventricular gangliocytoma.
